# Association of statin use with reduced risk of Alzheimer’s disease: findings in UK Biobank

**DOI:** 10.3389/fnagi.2026.1961033

**Published:** 2026-10-06

**Authors:** Hui Yang, Can Zhu, Jialu He, Yan Wei, Dongmei Li, Xuan Luo, Ying Wang

**Affiliations:** 1 Department of Clinical Research Center, Affiliated Hangzhou First People’s Hospital, Westlake University School of Medicine, Hangzhou, Zhejiang, China; 2 School of Pharmacy, Hangzhou Normal University, Hangzhou, Zhejiang, China; 3 School of Public Health Management, Hangzhou Normal University, Hangzhou, Zhejiang, China; 4 Laboratory of Medicine, Affiliated Hangzhou First People’s Hospital, Westlake University School of Medicine, Hangzhou, Zhejiang, China; 5 Zhejiang Key Laboratory of Zero Magnetic Medicine, Affiliated Hangzhou First People’s Hospital, School of Medicine, Westlake University, Hangzhou, Zhejiang, China

**Keywords:** Alzheimer’s disease, cognitive function, dementia, statins, UK Biobank

## Abstract

**Background:**

Previous studies on the association between statin use and the risk of Alzheimer’s disease (AD) have yielded inconsistent results. This study, based on the large-scale UK Biobank cohort, evaluates the relationship between statin use and the risk of new-onset AD, survival in patients with AD, and cognitive function.

**Methods:**

This study included 502,401 participants with a median follow-up of 9.24 years. The association between statin use and the risk of AD was analyzed using Cox proportional hazards models, logistic regression, and propensity score matching. The robustness of the results was assessed through internal validation using training and validation groups, as well as subgroup analyses.

**Results:**

A total of 975 new cases of AD were recorded. Multivariate Cox regression analysis showed that statin use was associated with a reduced risk of AD (HR = 0.72, 95% CI: 0.52–0.98; *p* < 0.05), and the results remained consistent after propensity score matching (*p* < 0.05). Among individuals aged ≥65 years, statin users demonstrated better cognitive performance than non-users. Concomitant use of antihypertensive or antidiabetic medications did not significantly alter the protective effect of statins.

**Conclusion:**

Statin use was associated with a modestly lower risk of AD and slower cognitive decline. These findings highlight the importance of enhanced clinical awareness in identifying AD patients and those at risk of adverse outcomes.

## 1 Introduction

Alzheimer’s disease (AD) is a progressive neurodegenerative disorder characterized by the accumulation of amyloid β (Aβ) plaques and hyperphosphorylated tau protein, leading to memory loss, cognitive decline, and impaired ability to perform daily activities (Lane et al., 2018; Twarowski and Herbet, 2023; Kalaria et al., 2008). As one of the most common causes of dementia (Livingston et al., 2024). AD represents a major public health challenge in the 21st century, and there are currently no effective methods to prevent or halt its progression.

Statins are widely prescribed for their significant lipid-lowering and cardiovascular protective effects and are the most commonly used cholesterol-lowering medications (Adhyaru and Jacobson, 2018; Gu et al., 2014; Mach et al., 2019). In recent years, the potential neuroprotective effects of statins have attracted increasing attention. In addition to lowering cholesterol, statins exert pleiotropic effects, including anti-inflammatory and antithrombotic actions, regulation of protein aggregation associated with neurodegenerative diseases, modulation of insulin signaling and effects on neurotransmitter activity (Choudhary et al., 2023; Mahmoudi et al., 2026; Barbiellini Amidei et al., 2021; Youn et al., 2024). These mechanisms are thought to potentially help improve cerebral perfusion and reduce neuronal damage. Cholesterol homeostasis is strictly regulated in the central nervous system. This suggests that statins may exert neuroprotective effects by influencing disease-related pathways beyond their cardiovascular indications. Given that high cholesterol levels in middle age are associated with subsequent cognitive decline and AD risk (Anstey et al., 2008; Wu et al., 2025). Statin therapy may represent potential therapeutic relevance in AD.

However, evidence regarding the effect of statins on the risk of AD varies significantly across different study designs. Systematic reviews and meta-analyses of randomized controlled trials (RCTs) report that statin use is not associated with cognitive impairment (Ott et al., 2015; McGuinness et al., 2016; Zhou et al., 2021), but a protective effect against the onset of AD has not yet been conclusively demonstrated. In contrast, the results from observational studies have been inconsistent. A cohort study indicates that statin drugs are significantly associated with a reduced risk of AD (Ren et al., 2024). Statin treatment does not pose any risk to neurocognition, and suggest that statins may have positive effects (Olmastroni et al., 2022; Royall and Palmer, 2024; Loera-Valencia et al., 2019). A longitudinal clinical-pathological study, however, reported no significant association between the two (Arvanitakis et al., 2008). Furthermore, some studies have shown that statin use may increase the risk of AD (Nedelec et al., 2023). This apparent contradiction likely stems from differences in study design, heterogeneity in population characteristics (such as age, sex, and race), variations in the lipophilicity of statins and the permeability of the blood–brain barrier, as well as the potential moderating effects of lifestyle and genetic factors (Olmastroni et al., 2022; Power et al., 2015; Titolo et al., 2025; Geifman et al., 2017). Furthermore, regulatory agencies such as the U.S. Food and Drug Administration (FDA) have issued warnings regarding potential non-serious and reversible cognitive adverse events, further highlighting the need for comprehensive evaluation (Ott et al., 2015).

Previous studies, particularly RCTs, have often struggled to detect long-term outcomes of AD due to relatively short follow-up periods and insufficient statistical power; observational studies, on the other hand, are prone to unmeasured confounding factors, such as indication bias and the healthy user effect. To address these shortcomings and overcome the limitations of previous evidence, this study aims to rigorously assess the association between statin use and the risk of cognitive impairment and AD using a large-scale population-based cohort that integrates genetic data. Unlike systematic reviews, we conducted a multilevel, multidimensional analysis of longitudinal data using standardized methods. We aim to elucidate whether statins can serve as a disease-modifying treatment for AD, thereby providing reliable empirical evidence to optimize clinical guidelines for AD prevention.

## 2 Methods

### 2.1 Study population

Participants for this study were drawn from the UK Biobank^1^, a large population-based prospective cohort study. Between 2006 and 2010, the UK Biobank recruited approximately 502,486 participants aged 40–69 years from England, Scotland and Wales (Sudlow et al., 2015). All subjects provide extensive demographic and health-related information and biospecimens and are followed up on an ongoing basis (Sudlow et al., 2015; Zhang et al., 2021). The UK Biobank received ethical approval from the National Health and Social Care Information Management Board and the National Health Service North West Multi-Centre Research Ethics Committee. Analyses were conducted under application number 41376. All participants gave informed consent by means of an electronic signature before joining the study. Participants with a diagnosis of AD at baseline were excluded. Participants without a diagnosis of AD at baseline, participants with complete data on medication use, and participants with adequate follow-up time were included in the study. Participants with missing data on key covariates were excluded. The data cut-off date for the analysis is 1 August 2023. Baseline data are shown in Supplementary Table S1.

### 2.2 Definition of outcome

The primary outcome of this study was incident AD, defined as a first diagnosis of AD occurring during the follow-up period. AD was identified using an algorithm that integrated diagnoses from multiple sources: hospital records (ICD-9; ICD-10), primary care data, death registries, hospitalization records (including participants’ self-reported medical conditions, medication use data) and self-reported medical conditions collected via touchscreen questionnaires during baseline and follow-up assessments. Participants with a prior diagnosis of AD recorded before the baseline assessment were excluded from the analysis. The use of an algorithm that integrates data from electronic health records and self-reports, without independent confirmation by biomarkers such as cerebrospinal fluid (Aβ/tau) or standardized neuropsychological assessments, may introduce misclassification of outcomes. This limitation is inherent in large-scale cohort studies that rely on routinely collected health data. In addition, cognitive test decline was used as a secondary outcome.

### 2.3 Statin use

Information about statins was collected at baseline through a touchscreen questionnaire, which was verified in verbal interviews and reported as a drug code. In addition, information on medication use from prescription records in primary care data and hospitalization records was included. Given that the number of people using simvastatin far exceeded the number using any other type of statin (Supplementary Table S3), we abandoned the approach of grouping by drug type. Statins use was categorized as follows: participants who took atorvastatin, fluvastatin, pravastatin, rosuvastatin, or simvastatin from the start of the study through the end of follow-up were classified as “statin users”; those who took other medications or had never been exposed to any medication were classified as “non-statin users.”

### 2.4 Cognitive measures

Lyall et al. (2016) provided a detailed description of the cognitive function tests administered to participants in the UK Biobank, including data missingness and internal variability over time. We extracted touchscreen-collected memory tests for longitudinal analysis: (1) reasoning/fluid intelligence test (FIT) (verbal and numerical reasoning, scored as number of correct answers out of 13 within 2 min), (2) trail making (Executive function is assessed using digit-to-digit and digit-letter alternating connection tasks; scores are based on completion time (in seconds), with longer times indicating poorer performance), (3) symbol digit substitution (Processing speed is assessed based on the number of correct symbol-to-number matches made within 60 s; a higher score indicates better information processing efficiency), and (4) numeric memory (working memory, scored as maximum number of digits correctly recalled in reverse order). Due to the insufficient amount of data for trail making, symbol digit substitution, and numeric memory, we fitted them with an algorithm, and the resulting data were abbreviated as general fluid test (GFT). We divided into two groups based on statin use or not and calculated risk scores using logistic regression. In addition, we performed principal component analysis (PCA) on these cognitive indicators to reduce the dimensionality of the downstream statistics (Fawns-Ritchie and Deary, 2020).

### 2.5 Covariates

The following covariates were measured at the baseline. Demographic variables included age (Field 21,022), sex (Field 31), socioeconomic variables included education, categorized as higher (college/university degree or other professional qualification) or lower (Field 6,138). Lifestyle variables included alcohol intake frequency (Field 1,558), and smoking status (Field 20,116). Other medication variables including antihypertensive use, and antidiabetic drug use, were from medication for cholesterol, blood pressure, diabetes, or take exogenous hormones (Field 6153/6177).

### 2.6 Statistical analysis

We summarized differences in baseline characteristics. Categorical variables were tested using chi-square tests, and continuous variables were tested using ANOVA. Follow-up time was calculated as the time from the last completed touchscreen questionnaire to the first diagnosis of AD, the date of death, the date of loss to follow-up, or the final date (in years) when admission information was available, whichever came first. To avoid statin use before the start of the study, we defined the time of the first occurrence of statin use as the start of the study. Cox proportional risk models were used to calculate the HR (risk ratio) and 95% CI. To address confounding by indication, we employed two complementary approaches: (1) multivariable adjustment for a comprehensive set of covariates including comorbidities and concomitant medications (antihypertensive and antidiabetic drugs), and (2) propensity score matching (PSM) to balance measured baseline characteristics between statin users and non-users. Model 1 was adjusted for demographic and temporal variables, and model 2 was further adjusted for socioeconomic, lifestyle, and medication variables (Figure 1) (Supplementary Table S4). The data were randomly partitioned into a test group (80%), for preliminary analysis and model development, and a validation group (20%), for validation of results. A restricted cubic spline (RCS) model was used to fit the nonlinear relationship between the relevant biomarkers and AD risk, and the inflection point was changed to a critical point, the default median. Subsequently, nomogram analyses were performed to predict the 5- and 10-year risk of AD onset according to model 2. Propensity score matching (PSM) was performed using a 1:1 nearest-neighbor matching algorithm with a caliper of 0.2 times the standard deviation of the propensity score, to improve study reliability and reduce confounding by indication. All statistical analyses were performed using R software version 4.2.1.

**Figure 1:**
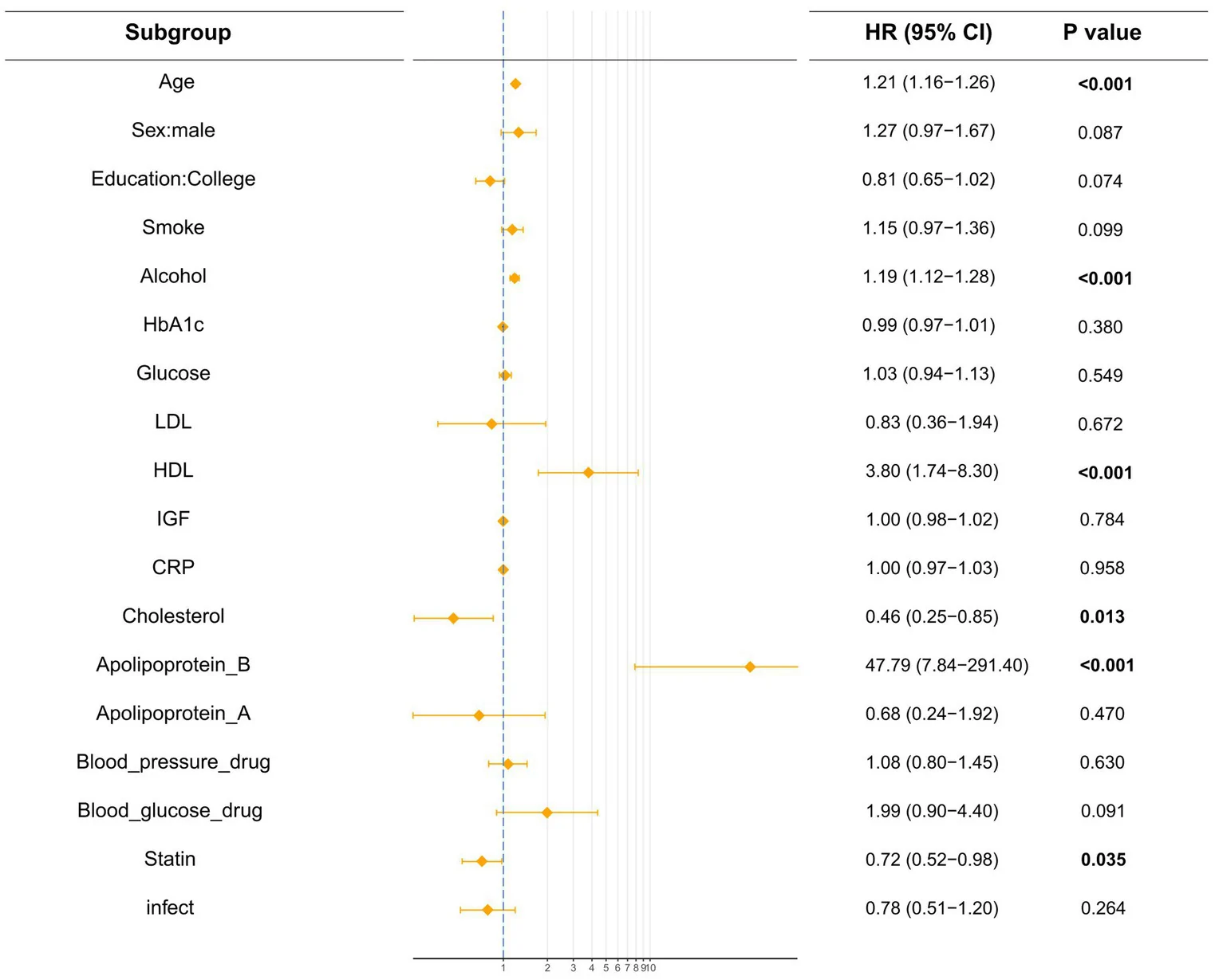
Forest plots for multi variable Cox analysis for risk of AD.

## 3 Results

### 3.1 Participant characteristics

This study included a total of 502,401 participants from the UK Biobank. The mean age was 56.53 years [standard deviation (SD): 8.05], and 54.41% were women (Supplementary Table S1). During a median follow-up period of 9.24 years, a total of 2,627 new cases of dementia were recorded, including 975 cases of AD (Supplementary Table S2). Compared with participants without AD, those with newly diagnosed AD tended to be older (mean age 64.5 years), had a higher proportion of women (51.28%), higher smoking rates, lower educational attainment, and were more likely to be taking antidiabetic and antihypertensive medications. Estimates of the association between statin use and AD risk during follow-up are shown in Supplementary Figure 1. There were significant differences in AD incidence between the statin treatment group and the unexposed group.

### 3.2 Association of statin use with reduced risk of AD

To investigate the association between statin use and the risk of AD, subgroups were defined by sex, age, smoking status, and educational level, and multivariately adjusted hazard ratios were estimated. In the full-sample multivariable logistic regression (Supplementary Table S5), statin use reached marginal statistical significance (HR 0.72, 95% CI 0.52–0.98) (Figure 1). Participants were randomly divided into a training group (80%) and a validation group (20%). In the training set, after multivariate adjustment, statin use showed a trend toward statistical significance (HR 0.76, 95% CI 0.52–1.10). The validation set also showed a similar trend (HR 0.63, 95% CI 0.36–1.11) (Supplementary Figure 1). Lower low-density lipoprotein (LDL) levels, lower apolipoprotein A (ApoA) levels, lower cholesterol levels and lower educational attainment also showed HR estimates below 1, but their confidence intervals were wide and included 1.0. Taken together, the data suggest a modest protective association between statin use and AD risk, although the precision varied by model specification. It is important to note that these findings are observational and may be influenced by residual confounding, including confounding by indication, despite our efforts to adjust for measured covariates.

### 3.3 Association of statins on survival in patients with AD

Based on the above predictors, we developed an AD risk prediction model (nomogram) to estimate the probability of survival over time and predict 5-year and 10-year survival rates (Figure 2). Each predictor corresponds to a specific score; the total score represents the predicted probability of developing AD. For any given subject, the higher the total score, the lower the probability of survival. A total score of 187 corresponded to predicted survival probabilities of 94.0 at 5 years and 92.5%% at 10 years. The ROC curves for training and validation sets were nearly identical (Figure 3), indicating good calibration. These results imply that statin use may be associated with longer dementia-free survival.

**Figure 2:**
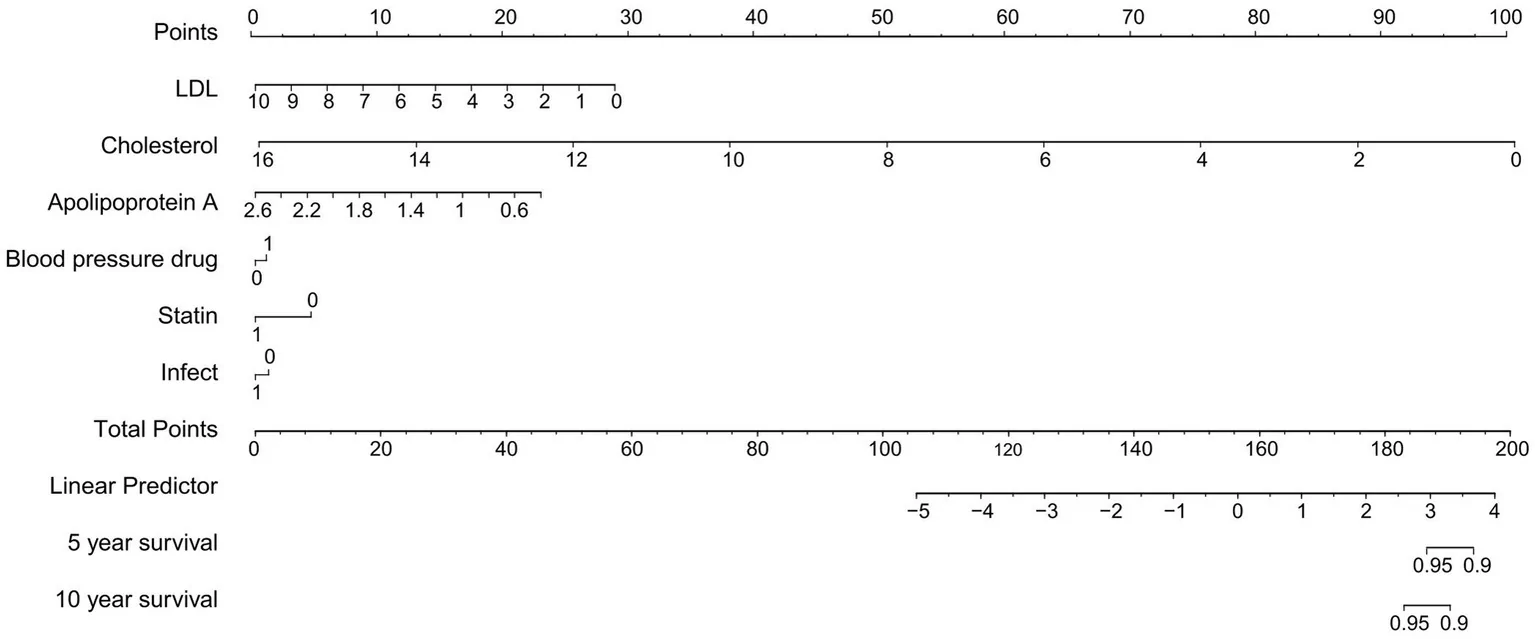
Construction of the nomo predictive model. Impact of statin use on 5- and 10-year survival in patients with AD.

**Figure 3:**
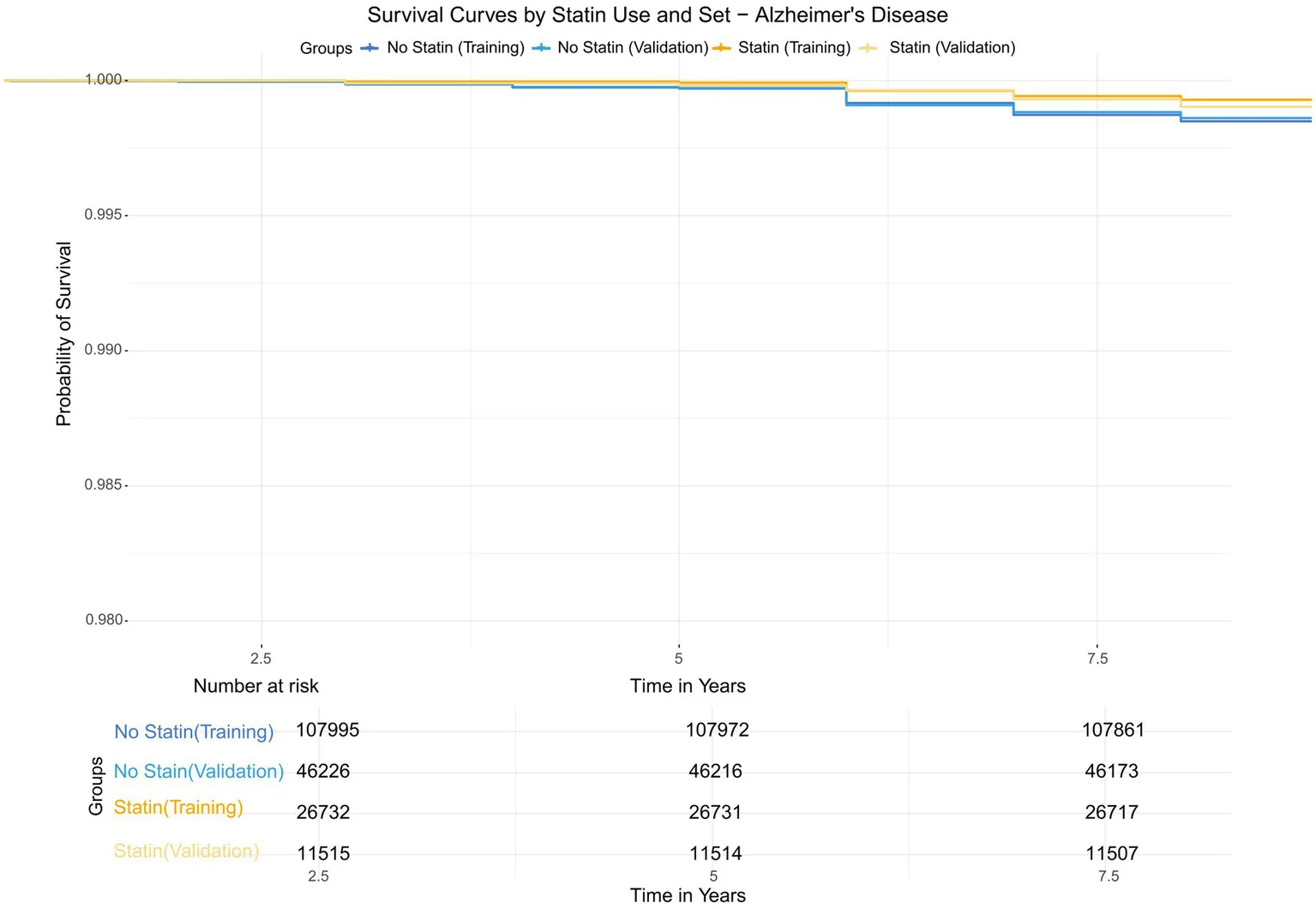
Predicted work characteristics (ROC) curves for AD. ROC curves, AUC, and area under the curve for the AD patient model in the training and validation cohorts.

### 3.4 Effect of statins use on cognitive performance

To investigate whether statin use affects cognitive function, we conducted a preliminary analysis and found no significant overall difference in cognitive ability between statin users and non-users (Supplementary Table S4). Given that age may be an important confounding factor, we divided participants into two groups based on age: < 65 years and ≥65 years. Among participants aged ≥65 years, those using statins had higher cognitive performance scores and lower risk scores compared with AD patients who did not use statins. Similar results were observed among patients with dementia (Table 1). This suggests that statin use may slow cognitive decline in older adults.

**Table 1:** Associations of statin use on cognitive performance.

		Statin users				Non-statin users			
Cognitive test		HR	95% CI	95% CI	*P*	HR	95% CI	95% CI	*P*
			Lower	Upper			Lower	Upper	
Model 1 Age ≥65									
AD	FIT	0.963	0.507	1.832	0.003	1.018	7.602	1.364	<0.001
	GFT	1.403	0.582	3.384	0.002	1.371	0.923	2.038	<0.001
Model 2 Age ≥65									
AD	FIT	0.888	0.459	1.718	0.008	0.873	0.618	1.232	<0.001
	GFT	1.466	0.604	3.557	0.001	1.728	1.150	2.597	<0.001
Model 1 Age <65									
AD	FIT	0.569	0.279	1.157	0.117	0.695	0.361	1.339	0.038
	GFT	1.771	0.877	3.578	<0.001	1.331	0.602	2.946	0.001
Model 2 Age <65									
AD	FIT	0.592	0.273	1.286	0.001	0.679	0.346	1.333	<0.001
	GFT	1.700	0.843	3.429	0.001	1.365	0.613	3.037	<0.001

Model 1: adjusted for age and sex.

Model 2: adjusted for age, sex, education, smoking, self-report, health status; and stratified by statin use or not. The following four assessment tests were chosen: reasoning/fluid intelligence test (FIT), trail making, symbol digit substitution, and numeric memory. Due to limited available data, the last three items were combined into a single group, referred to as the general fluid test (GFT).

### 3.5 Statin interactions with antihypertensive and antidiabetic drugs

Considering the impact of comorbidities, we evaluation included patients taking statins, antihypertensive agents, and antidiabetic agents concomitantly. In Cox univariate analysis, a antihypertensive (HR 2.04, 95% CI 1.75–2.39) and antidiabetic drugs (HR 3.16, 95% CI 2.39–5.47) were associated with increased AD risk, statins showed a protective effect (HR 0.42, 95% CI 0.35–0.50). In Cox multivariate analysis, only antidiabetic drugs remained a risk factor, though not statistically significant (HR 1.91, 95% CI 0.91–4.02). Antihypertensive drugs (HR 0.96, 95% CI: 0.73–1.26) and statins (HR 0.76, 95% CI: 0.59–0.99) were protective factors (Table 2). This indicates that, after correcting for the effects of blood pressure-lowering drugs and hypoglycemic drugs, the use of statins can reduce the risk of AD by approximately 23.9%. The protective effect of statins persisted after propensity score matching (*p* < 0.05) (Supplementary Table S5). However, these findings should be interpreted with caution, as residual confounding from unmeasured factors (e.g., disease severity, adherence, lifestyle changes over time) cannot be fully excluded.

**Table 2:** Association of statins with antihypertensives and antidiabetic agents on the risk of AD.

One-factor Cox analysis			
Variables	HR	*p* value	CI
Blood pressure drug	2.04	2.370E-19	1.75–2.39
Blood glucose drug	3.16	1.137E-09	2.39–5.47
Statin	0.42	2.03659E-21	0.35–0.5

Multi-factor Cox analysis						
Variables	Exp (coef.)	Lower 95	Upper 95	Se (coef.)	z	Pr (>|z|)
Blood pressure drug	0.957515	0.7263	1.2624	0.141039	−0.308	0.75822
Blood glucose drug	1.913234	0.9108	4.0189	0.378693	1.713	0.08667
Statin	0.760966	0.5874	0.9859	0.132105	−2.068	0.03866

## 4 Discussion

Our study aimed to investigate whether there is an association between statin use and the risk of new-onset Alzheimer’s disease (AD), and to analyze potential confounding factors related to age, sex, and concomitant use of cardiovascular medications. Our findings demonstrate that, among individuals aged 65 and older, statin use is consistently associated with a lower risk of new-onset AD, better preservation of cognitive function, and longer dementia-free survival. These associations remained robust (*p* < 0.05) after adjusting for antihypertensive and antidiabetic medications and performing propensity score matching. However, several methodological considerations warrant caution. First, the observed hazard ratio (HR 0.72, 95% CI 0.52–0.98) reflects a small effect size, and the upper limit of the confidence interval is very close to 1.0. Second, the high 5-year and 10-year survival probabilities predicted by the nomogram (>90%) reflect the low cumulative incidence of AD in the UK Biobank cohort (0.19% over 9.24 years), rather than a strong protective effect of statins. Taken together, these results suggest a modest protective association between statin use and the risk of Alzheimer’s disease. However, this should be viewed as a basis for generating new hypotheses rather than as evidence supporting the routine prescription of statins specifically for the prevention of dementia.

Our study reported a protective association between statin use and Alzheimer’s disease (AD), consistent with the findings of previous observational studies (Olmastroni et al., 2022; Petek et al., 2023; Sparks et al., 2006). In addition, this study further expanded on these findings by analyzing the interactions between statins and other classes of cardiovascular drugs. Notably, although antihypertensive and antidiabetic medications were positively associated with the risk of AD in univariate analyses (HR of 2.04 and 3.16, respectively), these effects were largely attenuated (antihypertensives: HR = 0.96) or became statistically insignificant (antidiabetics: HR = 1.91) after multivariate adjustment. These results suggest that the apparent risks associated with antihypertensive and antidiabetic drugs stem primarily from indication bias. Hypertension and type 2 diabetes (T2D) themselves are known risk factors for AD, rather than the drugs used to treat them. Importantly, after full adjustment, only statins retained an independent protective effect, further confirming their unique protective role. Nevertheless, these findings must be interpreted with caution, as the inherent risk of confounding by indication is a major limitation in any observational pharmacoepidemiologic study. In real-world practice, statin prescriptions are driven by cardiovascular risk profiles that are themselves linked to dementia, making it difficult to fully disentangle drug effects from baseline disease status. Therefore, while we report a robust statistical association, our study design cannot establish causality.

Statins reduce intracellular cholesterol synthesis by competitively inhibiting HMG-CoA reductase, a metabolic pathway closely associated with the core pathology of AD (Olmastroni et al., 2022; Petek et al., 2023). Imbalances in cholesterol homeostasis can directly affect the processing of β-amyloid (Aβ) and promote pathological changes in tau protein (Chew et al., 2020; Simons et al., 1998; Czuba et al., 2017). Clinical evidence further indicates that elevated levels of total cholesterol and low-density lipoprotein cholesterol (LDL-C) concentrations are associated with faster cognitive decline in patients with AD (Xue-Shan et al., 2016; Lee et al., 2020), suggesting that elevated cholesterol may be a risk factor for cognitive impairment. From a molecular perspective, the CYP46 gene, which encodes cholesterol 24-hydroxylase, not only regulates cholesterol clearance in the brain, but its variants have also been shown to influence Aβ burden and levels of phosphorylated tau protein, and are associated with susceptibility to late-onset sporadic AD (Papassotiropoulos et al., 2003). This provides genetic support for the involvement of cholesterol metabolism in the pathogenesis of AD. However, observational studies face significant confounding factors. Cholesterol levels in patients with late-onset dementia often show a downward trend, possibly due to reduced synthesis or metabolic disturbances (Kivipelto and Solomon, 2006; Reitz et al., 2008). This makes it difficult to directly infer a causal relationship between elevated cholesterol and AD risk.

Although cholesterol metabolism plays a key role in the progression of AD, the complexity of longitudinal data also highlights the importance of vascular factors. The neuroprotective potential of statins must be considered in light of their impact on the cerebrovascular system, particularly cerebral small vessel disease (CSVD). CSVD, which manifests as white matter hyperintensities (WMH) on brain imaging, is a progressive disorder affecting small vessels in the brain’s white matter and is a major contributor to vascular dementia and a significant confounder in dementia studies (Fresnais et al., 2023; Chojdak-Łukasiewicz et al., 2021). A recent meta-analysis by Fresnais and Fure found limited evidence from RCTs regarding statin treatment for CSVD (Fresnais and Fure, 2025), reporting a non-significant trend towards lower WMH volume (MD = −4.44, 95% CI −10.19–1.31). This underscores the complexity of the relationship between statins, vascular pathology, and cognition. In our study, the lack of detailed neuroimaging data on CSVD and WMH burden represents a significant unmeasured confounder. The protective association observed between statins and AD may also be partially mediated by their multifaceted effects on cerebral microvasculature, such as improved endothelial function and reduced microvascular inflammation (Albert et al., 2001). However, without imaging data, we cannot disentangle the direct anti-amyloid effects from the indirect vascular benefits in our cohort.

Previous studies have suggested that statins may impair β-cell function, reduce insulin sensitivity, and pose a potential risk of neurotoxicity (Betteridge and Carmena, 2016). Decreased glucose metabolism in the brain is closely associated with neurotoxic damage and cognitive decline (German and Liao, 2023). After adjusting for the use of antidiabetic medications, the protective effects of statins not only persisted but also remained stable. This suggests that, compared to blood pressure and blood glucose levels, lipid-lowering effects may be a key determinant of AD risk. Furthermore, after adjusting for multiple factors, the significant association between antihypertensive medications and AD risk disappeared. This suggests that this risk factor is primarily influenced by confounding factors such as age and vascular comorbidities. The latest neuropathological evidence indicates that antihypertensive medications are, in fact, significantly associated with a reduced ADNC burden, suggesting that their neuroprotective effects may depend on specific drug-class effects rather than simply the degree of blood pressure reduction (Abdulrahman et al., 2022; Ding et al., 2020). Regarding these conflicting results, the protective effects of statins do not depend on improvements in glucose metabolism but is more likely attributable to their multifactorial mechanisms, including the suppression of microglial inflammation, improvement of microvascular function, and promotion of Aβ clearance (Petek et al., 2023; Chiatto et al., 2026). The direct central benefits are sufficient to offset the negative effects of peripheral hyperglycemia. Furthermore, the hyperglycemic effect represents a slowly accumulating risk, whereas anti-inflammatory and vascular protective effects take effect more rapidly in middle-aged and older adults (Barbiellini Amidei et al., 2021). Therefore, in the long term, the net effect remains protective. In summary, the analysis of drug interactions ruled out confounding factors and monitoring bias, indicating that the protective effects of statins are independent of glucose regulation pathways. Their multifaceted neuroprotective effects possess sufficient physiological potency to offset metabolic side effects.

The protective effects of statins also vary across different populations. This study confirmed that age plays a significant moderating role. Statin use improved cognitive function in participants aged 65 and older, but had no effect or a slightly negative effect on working memory in participants younger than 65. This age-dependent pattern is consistent with previous analyses from the UK Biobank (Alsehli et al., 2020) and may reflect changes in cholesterol metabolism and blood–brain barrier permeability that occur with aging. Regarding gender differences, recent evidence suggests that men treated with statins have higher survival rates and experience more pronounced cognitive protection, which may be related to gender-specific cholesterol metabolism and the interaction between hormones and the mevalonate pathway. Importantly, the effects of statins appear to be modulated by the APOE genotype, with APOE carriers exhibiting more pronounced cognitive benefits (Dagliati et al., 2021). These findings collectively support a precision medicine approach to statin therapy for AD prevention.

While our findings are encouraging, they should not be interpreted as evidence for the immediate use of statins as a preventive therapy for AD in clinical practice. The observational nature of our study, combined with the potential for residual confounding, means that these results are best viewed as hypothesis-generating. Our findings support the conduct of future randomized clinical trials. Such trials should be prospectively stratified by age, sex, and APOE genotype to validate these observational associations and determine the optimal timing, regimen, and duration of statin therapy for neuroprotection. In the meantime, clinicians should continue to prescribe statins according to current cardiovascular guidelines, and the absence of cognitive decline should not be a barrier to initiating or continuing statin therapy in elderly patients.

## 5 Limitations

This study has several limitations. First, although we used multivariate adjustment and propensity score matching to address measured confounders, residual confounding (such as dietary habits, physical activity, family history of dementia, and other unmeasured factors like APOE genotype) and indication-related confounding (statin users may themselves have higher cardiovascular risk or complications such as hypertension or diabetes) may still influence the observed associations. Second, the UK Biobank lacks detailed data on statin type, dose, pharmacokinetics, and duration of treatment, which precludes dose–response analyses and comparisons among different statin formulations. Third, AD diagnoses were based on algorithmically linked electronic health records and self-reported data, without confirmation by gold standards such as CSF biomarkers or amyloid PET, and may therefore involve misclassification of outcomes; cognitive function was assessed using only a limited set of tests, and we may not have captured subtle cognitive changes or all domains of cognition. Fourth, UK Biobank participants are predominantly of white European descent, which limits the generalizability of the study results to other populations. In summary, the findings of this study should be considered hypothesis-generating rather than confirmatory evidence; clinical recommendations for the use of statins in the primary prevention of AD require validation through randomized controlled trials.

## 6 Conclusion

Our findings suggest that, in this large observational cohort study, statin use is associated with a reduced risk of AD. However, given that the statistical significance was only marginal and that observational data have inherent limitations—particularly the potential for residual confounding factors—these results should not be interpreted as clinical evidence supporting the routine prescription of statins for the prevention of dementia in older adults. Nevertheless, our findings do not support the U.S. FDA’s warning regarding clinically significant statin-associated cognitive impairment in this population. Future RCTs stratified by age, sex, and APOE genotype are needed to validate these observational findings and determine whether statins have a genuine neuroprotective effect.

## Data Availability

The original contributions presented in the study are included in the article/Supplementary material, further inquiries can be directed to the corresponding author.

## Footnotes

1
www.ukbiobank.ac.uk
