# Supplementary material for: Association of statin use with reduced risk of Alzheimer’s disease: findings in UK Biobank
Source: Front Aging Neurosci. 2026 Oct 6;18:1961033. doi: 10.3389/fnagi.2026.1961033 (PMC13680480; doi:10.3389/fnagi.2026.1961033)
Supplement: Supplementary file 1 [file Data_Sheet_1.pdf]

Title:

Association of statin use with reduced risk of Alzheimer's disease: Findings in UK Biobank

Supplementary Information

Fig. S1 Linear associations between statin use and risk of AD

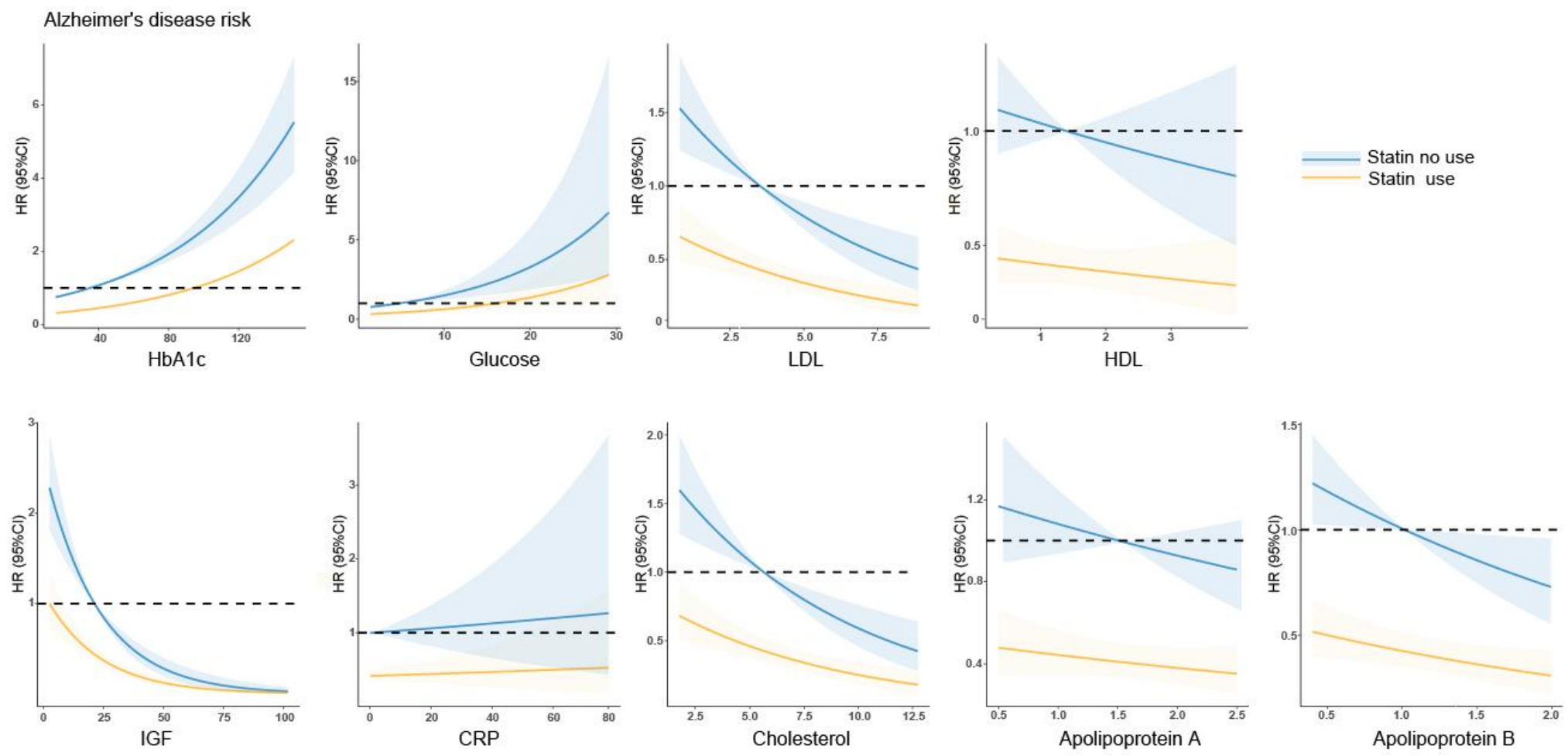

Fig. S2 Forest plots for multi variable Cox analysis for risk of AD.

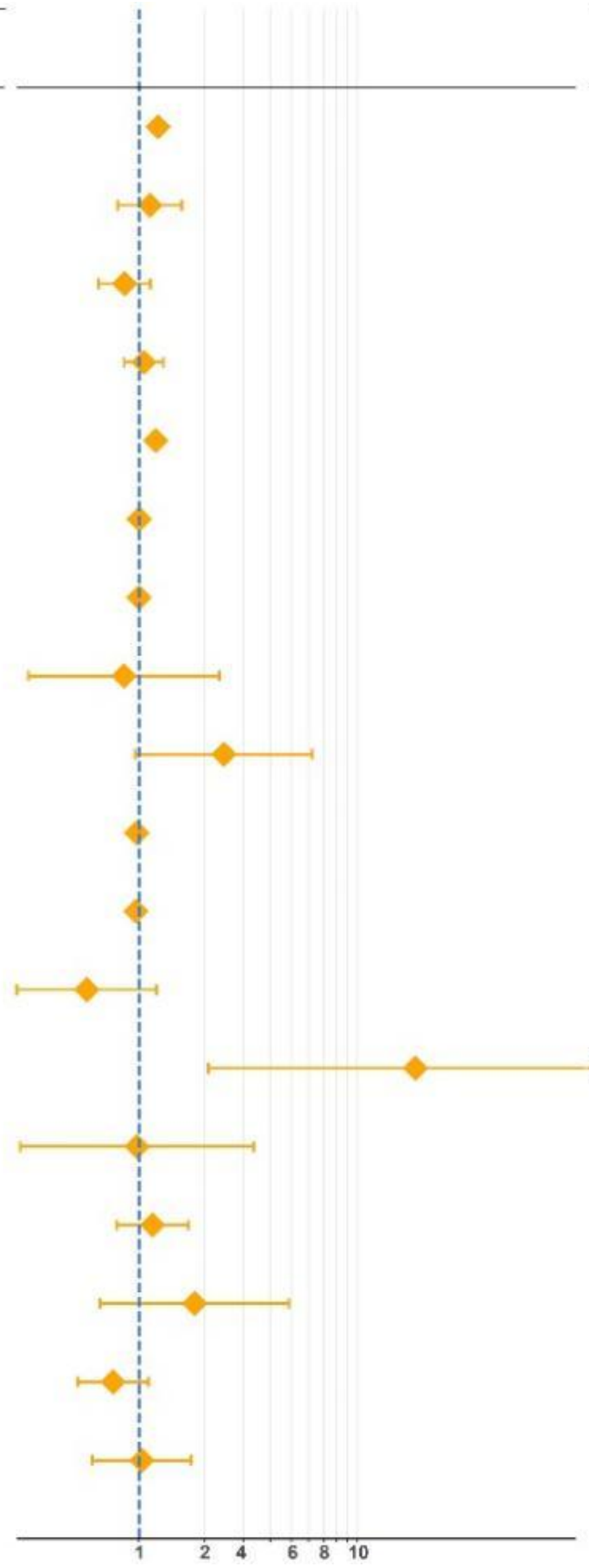

Subgroups	HR (95% CI)	P value
Age	1.22 (1.17−1.29)	**<0.001**
Sex:male	1.12 (0.80−1.57)	0.502
Education:College	0.86 (0.65−1.13)	0.276
Smoke	1.05 (0.86−1.29)	0.623
Alcohol	1.19 (1.10−1.30)	**<0.001**
HbA1c	1.00 (0.98−1.03)	0.818
Glucose	1.00 (0.89−1.12)	0.984
LDL	0.85 (0.31−2.34)	0.759
HDL	2.45 (0.96−6.24)	0.060
IGF	0.99 (0.97−1.02)	0.493
CRP	0.98 (0.94−1.03)	0.419
Cholesterol	0.58 (0.28−1.21)	0.143
Apolipoprotein B	18.44 (2.08−163.62)	**0.009**
Apolipoprotein A	0.98 (0.29−3.35)	0.975
Blood pressure drug	1.16 (0.79−1.69)	0.456
Blood glucose drug	1.80 (0.66−4.87)	0.248
Statin	0.76 (0.52−1.10)	0.151
Infect	1.03 (0.61−1.74)	0.906

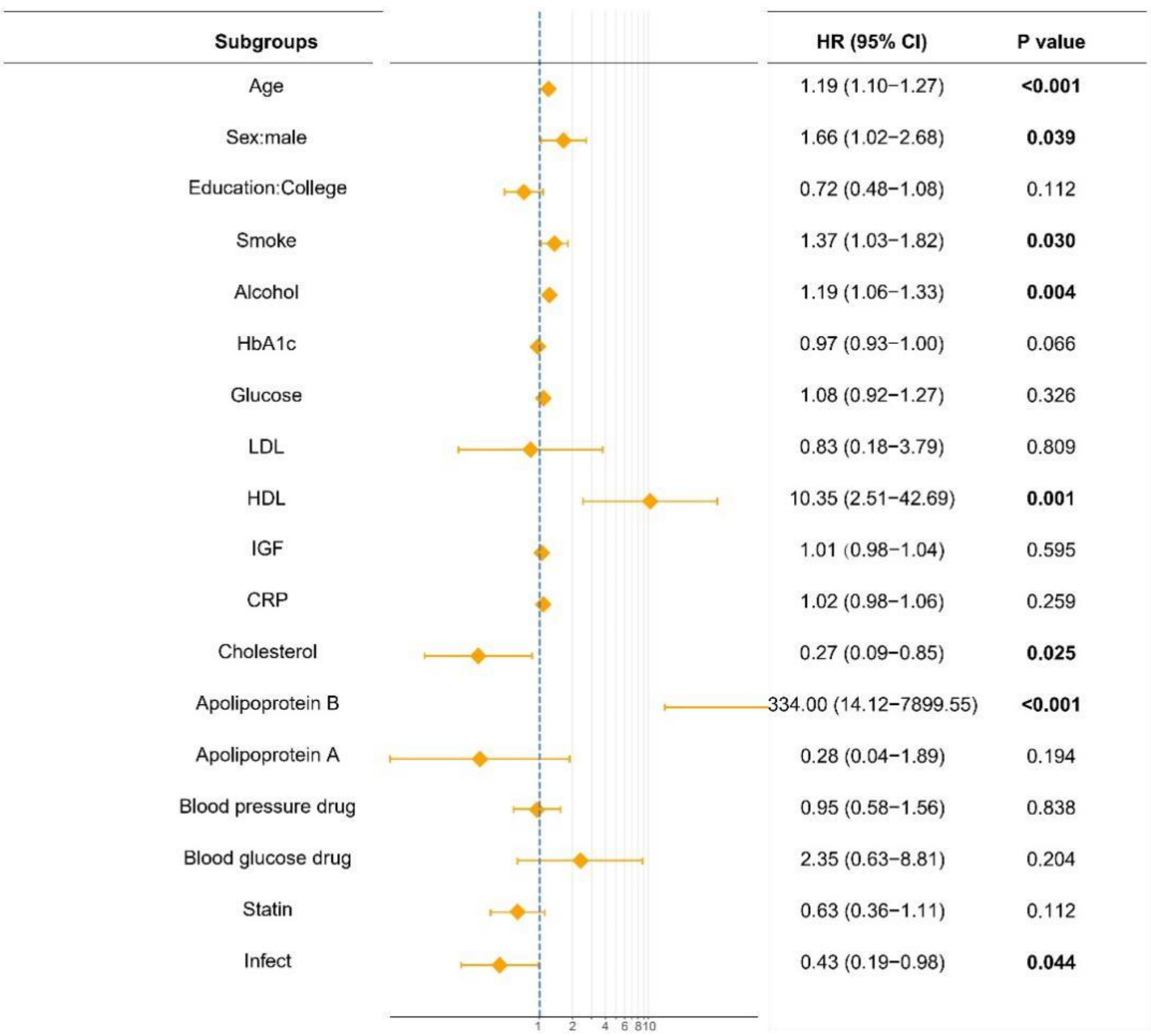

Table S1 Baseline characteristics of study participants

**Characteristic**	**All participants (n=502401)**	**No incident AD (n=501426)**	**Incident AD (n=975)**
**Age(SD)**	56.53(8.09)	56.51(8.09)	64.5(4.39)
**Sex(%)**			
Male	229063(45.59)	228563(45.58)	475(48.72)
Female	273338(54.41)	272863(54.42)	500(51.28)
**Education**			
College degree	161134(39.59)	160958(39.6)	176(30.4)
No college degree	245896(60.41)	245493(60.4)	403(69.6)
**Smoking status，n(%)**			
Current	52969(10.61)	52875(10.61)	454(47.29)
Previous	173014(34.64)	172602(34.62)	2 94(9.79)
Never	273471(54.75)	273017(54.77)	412(42.92)
**Alcohol status，n(%)**			
Daily or almost daily	101735(20.31)	101583(20.32)	164(16.91)
Three or four times a week	115429(23.04)	115231(23.05)	4 79(8.14)
Once or twice a week	129274(25.81)	129040(25.81)	234(24.12)
One to three times a	55843(11.15)	55764(11.15)	143(14.74)

month			
Special occasions only	57997(11.58)	57854(11.57)	152(15.67)
Never	40622(8.11)	40458(8.09)	198(20.41)
**HbA1c**	36.13(6.78)	36.13(6.77)	38.42(9.61)
**Glucose**	5.12(1.24)	5.12(1.24)	5.35(1.55)
**LDL**	3.56(0.87)	3.56(0.87)	3.41(0.95)
**HDL**	1.45(0.38)	1.45(0.38)	1.43(0.4)
**IGF**	21.4(5.70)	21.4(5.7)	19.89(5.75)
**CRP**	2.6(4.36)	2.6(4.36)	2.79(4.94)
**Cholesterol**	5.69(1.14)	5.69(1.14)	5.5(1.27)
**Apolipoprotein A**	1.54(0.27)	1.54(0.27)	1.01(0.24)
**Apolipoprotein B**	1.03(0.24)	1.03(0.24)	1.53(0.28)
**Blood pressure drug**	56062(11.16)	55863(11.14)	199(20.41)
**Blood glucose drug**	3344(0.67)	3321(0.66)	23(2.36)

Data presented as mean (SD) for continuous variables and number (%) for categorical variables.

P-values are derived using either Student's t-test or Chi-square test.

UKB:UK Biobank, AD: Alzheimer's diseases

Table S2 Comparing the relationship between statin use and the development of Alzheimer's disease and dementia

	Total	Statin users	Non-statin users
**All participants**	502401	142716	359685
**Alzheimer's disease**	975	139	836
**Dementia**	2627	325	2302

Excluding Alzheimer's disease/dementia diagnosed before 2006.

Table S3 Classification of Statins

Simvastatin	Atorvastatin	Fluvastatin	Pravastatin	Rosuvstatin
141973	504	6	87	146

Table S4 Associations of statin use on cognitive performance

**Model 1 Table whole cohort**										
**Statin users**						**Non-statin users**				
		**HR**	**95% CI Lower**	**95% CI Upper**	**P**	**HR**	**95% CI Lower**	**95% CI Upper**	**P**
**A**	FIT	0.752	0.470	1.205	<0.001	0.950	0.727	1.240	<0.001
**D**	GFT	1.567	0.898	2.735	<0.001	1.359	0.953	1.937	<0.001

**Model 2**									
**Statin users**					**Non-statin users**				
		**HR**	**95% CI Lower**	**95% CI Upper**	**P**	**HR**	**95% CI Lower**	**95% CI Upper**	**P**

**A**	FIT	0.745	0.458	1.212	0.003	0.824	0.606	1.12	<0.001
**D**	GFT	1.576	0.901	2.755	<0.001	1.626	1.135	2.331	<0.001

Model 1: adjusted for age and sex;

Model 2: adjusted for age, sex, education, smoking, self-report, health status; and stratified by statin use or not. The following four assessment tests were chosen: reasoning/fluid intelligence test(FIT), trail making, symbol digit substitution, and numeric memory. Due to limited available data, the last three items were combined into a single group, referred to as the general fluid test (GFT).

Table S5 Propensity score

**AD**	**HR**	**95% CI Lower**	**95% CI Upper**	**P**
Blood pressure drug	0.9981744	0.7635903	1.304825	<0.05
Blood glucose drug	0.9908376	0.5419710	1.811461	<0.05
Statin	0.9881163	0.7586837	1.286931	<0.05

cox analytics risk score

**Alzheimer's disease**	**Exp(coef)**	**Exp(-coef)**	**lower**	**CI**
**Blood pressure drug**	1.0097	0.9904	0.8358	1.220
**Blood glucose drug**	0.9834	1.0169	0.6423	1.506
**Statin**	0.9894	1.0107	0.8207	1.193
